# HIF-1*α*-Mediated miR-623 Regulates Apoptosis and Inflammatory Responses of Nucleus Pulposus Induced by Oxidative Stress via Targeting TXNIP

**DOI:** 10.1155/2021/6389568

**Published:** 2021-08-03

**Authors:** Xiaogang Bao, Zhenhua Wang, Qi Jia, Sibo Shen, Likang Wu, Qi Jiang, Changwei Li, Guohua Xu

**Affiliations:** ^1^Department of Orthopedic Surgery, Spine Center, Shanghai Changzheng Hospital, The Second Military Medical University, Shanghai, China; ^2^Department of Laboratory Medicine, Shanghai Changzheng Hospital, The Second Military Medical University, Shanghai, China; ^3^Department of Orthopedic Oncology, Spine Tumor Center, Shanghai Changzheng Hospital, The Second Military Medical University, shanghai, China; ^4^Hebei Key Laboratory of Active Components and Functions in Natural Products, College of Chemical Engineering, Hebei Normal University of Science and Technology, Qinhuangdao, 066600 Hebei, China; ^5^School of Pharmaceutical Engineering & Life Science, Changzhou University, Changzhou, Zhejiang, China; ^6^Department of Orthopedic Surgery, Changhai Hospital, The Second Military Medical University, Shanghai, China; ^7^Shanghai Key Laboratory for the Prevention and Treatment of Bone and Joint Diseases with Integrated Chinese-Western Medicine, Shanghai Institute of Traumatology and Orthopedics, Rui Jin Hospital, Shanghai Jiao Tong University School of Medicine, Shanghai, China

## Abstract

Excessive apoptosis and inflammatory responses of nucleus pulposus (NP) cells induced by oxidative stress contribute to intervertebral disc degeneration (IVDD). Though some microRNAs are associated with IVDD, the specific microRNA that can mediate apoptotic and inflammatory responses of NP cells induced by oxidative stress synchronously still needs further identification. Here, we find that microRNA-623 (miR-623) is downregulated in IVDD and its expression is regulated by hypoxia-inducible factor-1*α* (HIF-1*α*) under oxidative stress conditions. Mechanistically, HIF-1*α* is observed to promote miR-623 expression by directly binding to its promoter region (−1,994/−1,987 bp). Functionally, miR-623 is found to work as an intermediator in alleviating apoptosis and inflammatory responses of NP cells induced by oxidative stress via regulating thioredoxin-interacting protein (*TXNIP*) expression by directly targeting its 3′-untranslated region (3′-UTR). Thus, on elucidating the expression and functional mechanisms of miR-623, our study suggests that miR-623 can be a valuable therapeutic target for treating oxidative stress-induced IVDD.

## 1. Introduction

Intervertebral disc degeneration (IVDD) is a common spinal disease, which is typically manifested by lower back pain and reduced lumbar spine support [1] The intervertebral disc (IVD) has been elucidated to consist of three interrelated structures: the nucleus pulposus (NP), annulus fibrosus (AF), and cartilaginous endplate. As NP forms the inner core of IVD, it has been shown to preserve high water content; thereby, allowing the vertebral disc to sustain forces of compression and torsion [2]. Many studies have shown that excessive apoptosis and inflammatory responses of NP cells trigger metabolic disorders, downregulate extracellular matrix (ECM) production, and abolish conventional homeostatic tissue remodeling, which eventually lead to IVDD [3, 4]. Thus, determining key molecular mechanisms of NP cells that regulate apoptosis and inflammatory responses can significantly aid in managing IVDD.

Among many factors that can cause IVDD, accumulation of reactive oxygen species (ROS) has been reported to be one of them [5, 6]. Recent findings have proved the presence of oxidative stress and escalated concentrations of oxidation products in degenerated discs. Furthermore, reports have confirmed that oxidative stress and subsequent mitochondrial dysfunction play a role in facilitating intrinsic cellular apoptosis in NP cells [3]. Moreover, ROS has been shown to activate signaling pathways such as mitogen-activated protein kinases (MAPKs) and the nuclear factor kappa-light-chain-enhancer of activated B cells (NF-*κ*B) to promote expression of ECM proteases and proinflammatory genes, while inhibiting expression of ECM-related genes and anticatabolic genes [7]. Thus, establishing key molecular mechanisms of apoptosis and inflammatory responses in oxidative stress-induced NP cells can assist in effectively managing IVDD.

MicroRNAs (miRNAs) are 20–22 nucleotide long base sequences that can target 3′-untranslated regions (3′-UTR) of the target gene and thereby inhibit its expression. Increasing evidences have revealed the influence of miRNAs in IVDD such as mediating apoptosis and inflammatory responses of NP cells. miR-155 is a well-known miRNA that has been shown to play a crucial role in regulating apoptotic pathways. Compared to control NP cells, miR-155 expression has been reported to be significantly downregulated in IVDD and overexpression of miR-155 in NP cells has been shown to inhibit apoptosis by suppressing the expression of FAS-associated death domain protein (FADD) and caspase-3 [8]. Similarly, many studies have revealed the downregulation of miR-146a in IVDD. Regulated expression of miR-146a has been shown to significantly attenuate interleukin 1*β*- (IL-1*β*-) induced expression of tumor necrosis factor *α* (TNF-*α*), IL-6, matrix metalloproteinases (MMPs), and inducible nitric oxide synthase (iNOS) [9]. In addition, miR-145 overexpression has been demonstrated to attenuate apoptosis induced by oxidative stress and increase matrix synthesis in NP cells [10]. Thus, identifying the specific microRNAs that can comediate apoptotic and inflammatory responses of NP cells induced by oxidative stress may provide a potential therapeutic target for treating IVDD.

In the present study, we found that miR-623 was downregulated in IVDD and its expression was regulated by hypoxia-inducible factor 1*α* (HIF-1*α*) under oxidative stress conditions. Furthermore, miR-623 was observed to act as an intermediator in apoptosis and inflammatory responses of oxidative stress-induced NP cells by targeting thioredoxin-interacting protein (*TXNIP*) mRNA. Overall, our study proposes miR-623 as a potential therapeutic target for treating oxidative stress-induced IVDD.

## 2. Results

### 2.1. Oxidative Stress Downregulates miR-623 Expression in NP Cells

**Figure 1:**
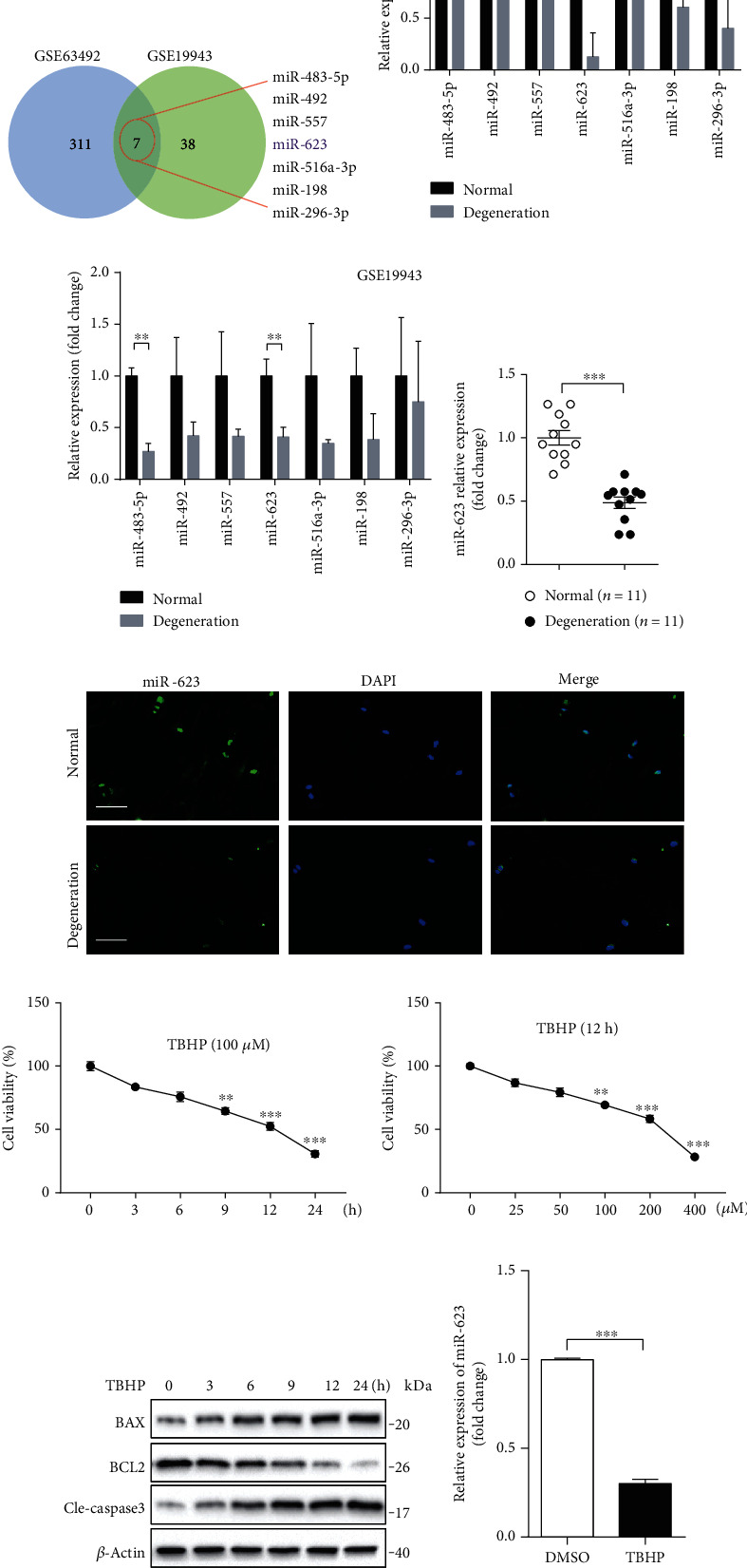
Oxidative stress dampens miR-623 expression in NP cells. (a) Differentially expressed miRNAs between normal and degenerated NP tissues were determined by analysis with microarray datasets of GSE63492 and GSE19943. Seven miRNAs were found to be downregulated in degenerated NP tissues both in the datasets of GSE63492 and GSE19943. (b) The fold change of relative mRNA expression of seven downregulated miRNAs (a) analyzed by the dataset of GSE63492 was shown as the column chart. (c) The fold change of relative mRNA expression of seven downregulated miRNAs (a) analyzed by the dataset of GSE19943 was shown as the column chart. (d) Quantification analysis of miR-623 mRNA expression in normal NP tissues (*n* = 11) and degenerated NP tissues (*n* = 11). (e) Immunostaining analysis of miR-623 mRNA expression in normal NP tissues and degenerated NP tissues. Scale bars represent 50 *μ*m. (f) The survival rate of NP cells incubated with 100 *μ*M TBHP for different hours. (g) The survival rate of NP cells incubated with different doses of TBHP for 12 hours. (h) The protein of BAX, BCL2, and cleaved caspase-3 expression in NP cells induced by 100 *μ*M TBHP for different hours. (i) Quantification analysis of miR-623 mRNA expression in NP cells with or without TBHP stimulation. ^∗^*P* < 0.05, ^∗∗^*P* < 0.01, and ^∗∗∗^*P* < 0.001. *P* values were analyzed by two-tailed *t*-tests in (b, c, d, and i) and one-way ANOVA in (f, g).

To identify the potential miRNAs that are involved in apoptosis and inflammatory responses of ROS-induced NP cells, we analyzed differentially expressed miRNAs in control and degenerated NP tissues using microarray datasets (GSE63492 and GSE19943) that were obtained from the Gene Expression Omnibus (GEO) database. Analyses of both the datasets revealed seven miRNAs that were downregulated in degenerated NP tissues (Figure 1(a)). To determine the most evidently downregulated miRNA, we compared the expression of all seven miRNAs in each dataset. Results showed that miR-623 expression had the highest significant difference (Figures 1(b) and 1(c)). Furthermore, to confirm the database-oriented results, miR-623 expression was evaluated in degenerated NP tissues from patients with IVDD and in healthy NP tissues from patients with spinal tumor. Quantitative real-time PCR (qRT-PCR) analysis revealed that miR-623 expression was significantly downregulated in NP cells of IVDD tissues (Figure 1(d)). Further, we verified the dampened expression of miR-623 in degenerated NP tissues by RNA fluorescence in in situ hybridization (RNA-FISH) analysis (Figure 1(e)).

On confirming the downregulated expression of miR-623 in IVDD, we then investigated whether miR-623 modulation was a response to ROS. Tert-butyl hydroperoxide (TBHP) was used as an exogenous ROS donor in previously published methodologies [11]. As shown in Figures 1(f)–1(h), stimulated primary NP cells with 100 *μ*M TBHP for 12 hours significantly induced NP cell apoptosis, as demonstrated by the decreased cell survival rate, decreased antiapoptotic regulator B-cell lymphoma 2 (BCL2) expression, and increased expression of BCL2-associated X (BAX). Meanwhile, miR-623 expression was found to be significantly dampened in the presence of 100 *μ*M TBHP stimulation for 12 hours (Figure 1(i)). Overall, our results demonstrated that miR-623 expression is downregulated in NP cells of IVDD which indicated that miR-623 might be involved in oxidative stress-induced apoptosis and inflammatory responses of NP cells.

### 2.2. miR-623 Attenuates Apoptosis of NP Cells Induced by Oxidative Stress

**Figure 2:**
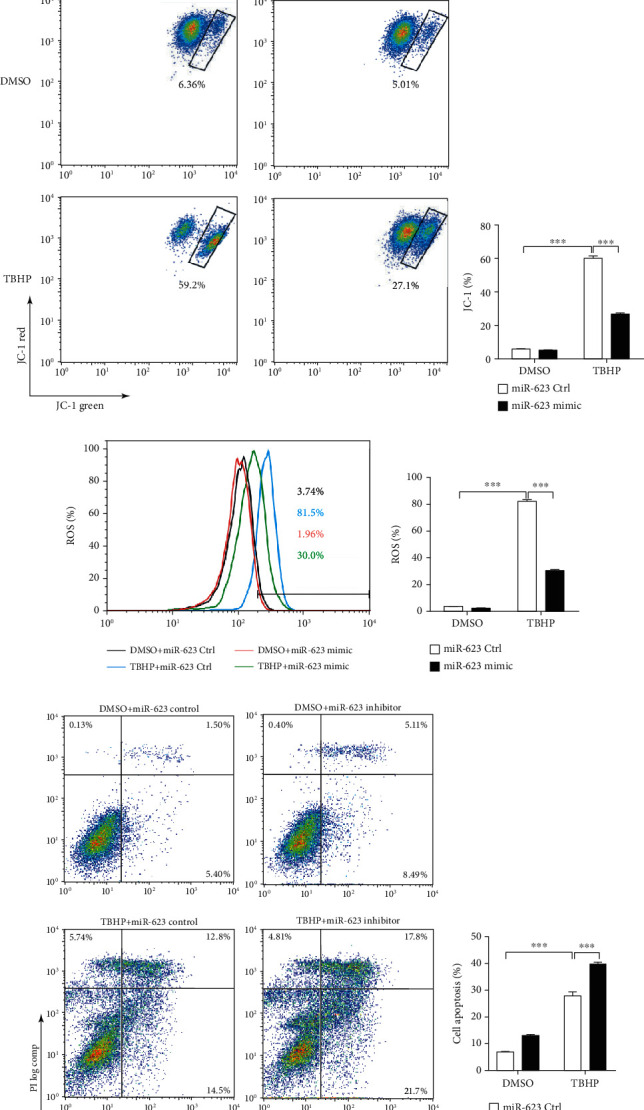
miR-623 decreases the apoptosis of NP cells induced by oxidative stress. (a) Quantification analysis of miR-623 mRNA expression in NP cells with or without miR-623 mimic transfection. (b) FACS analysis of NP cell apoptosis induced by TBHP with or without miR-623 mimic transfection. (c) The means of cell apoptosis in (b) were shown as the column chart. (d) Western blot analysis of BAX, BCL2, cleaved caspase-3, collagen II, and MMP13 expression in NP cells induced by TBHP with or without miR-623 mimic transfection. (e) FACS analysis of JC-1-positive cells induced by TBHP with or without miR-623 mimic transfection. (f) The means of JC-1-positive cells in (e) were shown as the column chart. (g) FACS analysis of ROS-positive cells induced by TBHP induced with or without miR-623 mimic transfection. (h) The means of ROS-positive cells in (g) were shown as the column chart. (i) FACS analysis of NP cell apoptosis induced by TBHP with or without miR-623 mimic transfection. (j) The means of cell apoptosis in (i) were shown as the column chart. (k) FACS analysis of JC-1-positive cells induced by TBHP with or without miR-623 mimic transfection. (l) The means of JC-1-positive cells in (k) were shown as the column chart. (m) FACS analysis of ROS-positive cells induced by TBHP induced with or without miR-623 mimic transfection. (n) The means of ROS-positive cells in (m) were shown as the column chart. ^∗∗∗^*P* < 0.001. *P* values were analyzed by two-tailed *t*-tests in (a) and two-way ANOVA in (c, f, h, j, l, and n).

To determine the role of miR-623 in NP cell apoptosis induced by oxidative stress, primary human NP cells were transfected with the miR-623 mimic or inhibitor before being treated with TBHP. As illustrated in Figures 2(a)–2(c), fluorescence-activated cell sorting (FACS) analysis revealed that miR-623 overexpression restricts TBHP-induced apoptosis of NP cells. Consistently, Western blot analysis revealed that miR-623 overexpression significantly restores the expression of BAX, cleaved caspase 3, MMP13, BCL2, and collagen II, which were induced or reduced, on TBHP treatment (Figure 2(d)). Studies have shown that mitochondria are not only the main source for cellular ROS but also specifically susceptible to oxidative stress-related damage [12]. JC-1 staining revealed that miR-623 significantly alleviates TBHP-decreased mitochondrial membrane potential (Figures 2(e)–2(f)). This suggested that miR-623 overexpression may protect mitochondria from oxidative stress-related damage. Similarly, C11 fluorescent dye-mediated detection of the mitochondrial ROS level revealed that miR-623 overexpression markedly restricts ROS production in TBHP-induced NP cells (Figures 2(g) and 2(h)). In contrast to the ameliorated role of miR-623 overexpression in TBHP-induced apoptosis and ROS production, miR-623 inhibitor transfection significantly promoted the apoptosis and ROS production of NP cells induced by TBHP (Figures 2(i)–2(n)). Overall, these results demonstrated that miR-623 attenuates NP cell apoptosis induced by oxidative stress.

### 2.3. miR-623 Inhibits Inflammatory Responses of NP Cells Induced by Oxidative Stress

**Figure 3:**
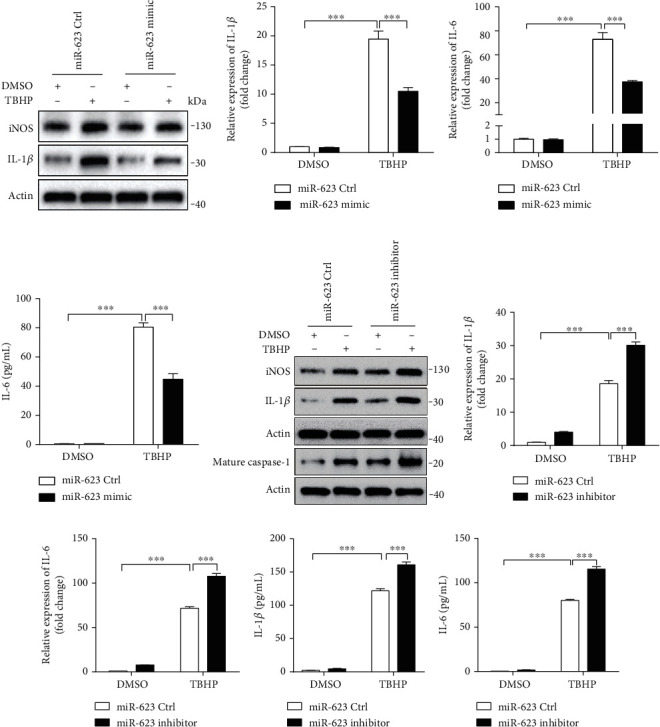
miR-623 inhibits the inflammatory responses of NP cells induced by oxidative stress. (a) Western blot analyses of IL-1*β* and iNOS in NP cells induced by TBHP with or without miR-623 mimic transfection. (b, c) Quantification analysis of IL-1*β* and IL-6 mRNA expression in NP cells induced by TBHP with or without miR-623 mimic transfection. (d) Quantification analysis of the secretion of IL-6 in culture medium of NP cells induced by TBHP with or without miR-623 mimic transfection. (e) Western blot analyses of IL-1*β*, iNOS, and cleaved caspase-1 (C-caspase-1) in NP cells induced by TBHP with or without miR-623 inhibitor transfection. (f, g) Quantification analysis of IL-1*β* and IL-6 mRNA expression in NP cells induced by TBHP with or without miR-623 inhibitor transfection. (h, i) Quantification analysis of the secretion of IL-1*β* and IL-6 in culture medium of NP cells induced by TBHP with or without miR-623 inhibitor transfection. ^∗∗∗^*P* < 0.001. *P* values were analyzed by two-way ANOVA.

On elucidating the antiapoptotic role of miR-623, we further investigated whether miR-623 mediates inflammatory responses in oxidative stress-induced NP cells. Gain-of-function experiments revealed that on transfection of miR-623 mimic, expressions of inflammatory markers such as iNOS, IL-1*β*, and IL-6 were significantly reduced in TBHP-treated NP cells (Figures 3(a)–3(d)). Conversely, loss-of-function experiments demonstrated that the miR-623 inhibitor significantly augmented the effect of TBHP on iNOS, IL-1*β*, and IL-6 expression (Figures 3(e)–3(i)). Thus, these results demonstrated that miR-623 inhibits inflammatory responses in NP cells induced by oxidative stress.

### 2.4. miR-623 Attenuates TBHP-Induced Apoptosis and Inflammation by Targeting *TXNIP* mRNA

**Figure 4:**
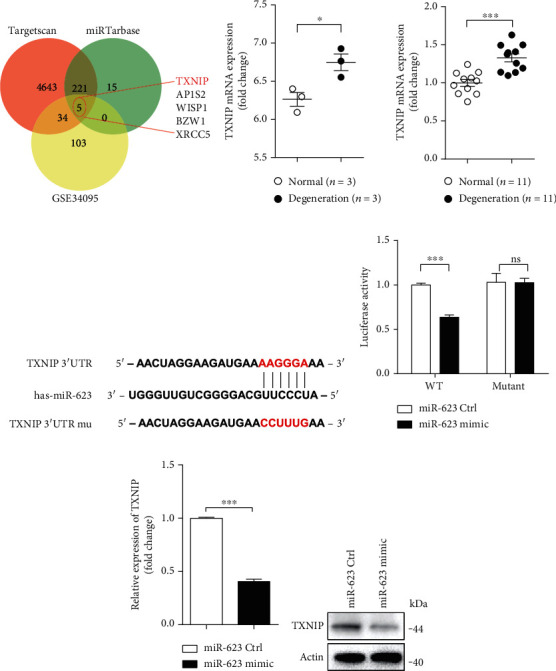
miR-623 inhibits TXNIP expression by targeting its 3′-UTR. (a) Bioinformatics analysis was performed to determine the predicted downstream targets of miR-623 with the dataset of TargetScan, miRTarBase, and GSE34095. (b) The fold change of relative mRNA expression of TXNIP analyzed by the dataset of GSE63492. (c) Quantification analysis of TXNIP mRNA expression in normal (*n* = 11) and degenerated (*n* = 11) NP tissues. (d) Schematic representation of the binding between miR-623 and the TXNIP 3′-UTR. (e) Relative luciferase activities were analyzed in 293T cells cotransfected with TXNIP 3′-UTR (wild-type or mutant) reporter plasmid and miR-623 mimics or miR-623 control. (f, g) qRT-PCR and Western blot analysis of TXNIP expression in NP cells transfected with miR-623 mimics or miR-623 control. ^∗^*P* < 0.05 and ^∗∗∗^*P* < 0.001. *P* values were analyzed by two-tailed *t*-tests in (a, b, d, and e).

**Figure 5:**
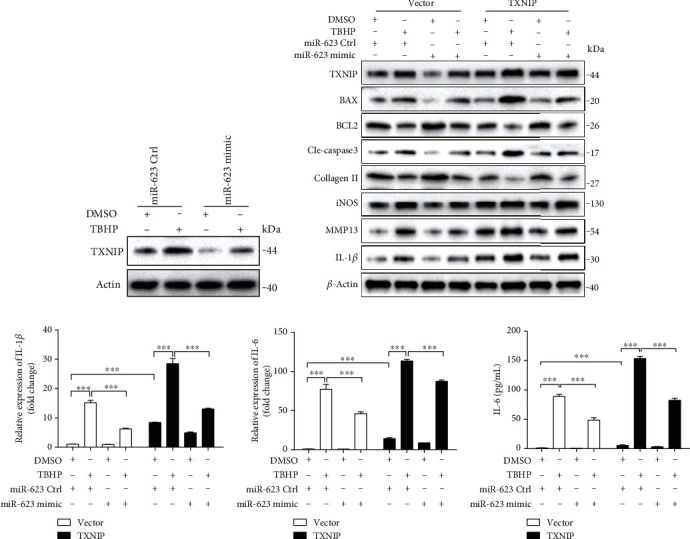
miR-623 attenuates TBHP-induced apoptosis and inflammation via TXNIP. (a) Western blot analysis of TXNIP expression in NP cells induced by TBHP with or without miR-623 mimic transfection. (b) Western blot analyses of TXNIP, BAX, BCL2, cleaved caspase-3, collagen II, iNOS, MMP13, and IL-1*β* in NP cells induced by TBHP with or without TXNIP overexpression and/or miR-623 mimic transfection. (c, d) Quantification analysis of IL-1*β* and IL-6 mRNA expression in NP cells induced by TBHP with or without TXNIP overexpression and/or miR-623 mimic transfection. (e) Quantification analysis of the secretion of IL-6 in culture medium of NP cells induced by TBHP with or without TXNIP overexpression and/or miR-623 mimic transfection. ^∗∗∗^*P* < 0.001. *P* values were analyzed by two-way ANOVA.

To identify the downstream targets of miR-623, bioinformatics analysis was performed using two miRNA databases, TargetScan and miRTarBase. Subsequently, 226 genes were selected for further investigation. On analyzing the microarray dataset, GSE34095, mRNA expressions of *TXNIP*, adaptor protein-1 complex subunit sigma-2 (*AP1S2*), WNT1-inducible-signaling pathway protein-1 (*WISP1*), basic leucine zipper and W-2 domain containing protein 1 (*BZW1*), and X-ray repair cross-complementing protein 5 (*XRCC5*) were found to be upregulated in IVDD among the selected 226 genes (Figures 4(a) and 4(b)). TXNIP has been reported as a key regulatory protein in oxidative stress-induced apoptosis and inflammatory responses [13]. Subsequently, we hypothesized that *TXNIP* might be the mRNA target of miR-623. To evaluate this hypothesis, we first compared the TXNIP expression level in normal IVDs and IVDD tissues. Results showed that TXNIP expression was upregulated in IVDD (Figure 4(c)). Further, the predicted binding sites of miR-623 on 3′-UTR of *TXNIP* were shown in Figure 4(d). To verify these putative binding sites, site-directed mutagenesis was performed (Figure 4(d)). 293T cells were transfected with wild-type (WT) or mutated 3′-UTR constructs of *TXNIP*, and luciferase activity was measured following cotransfection with miR-623. As shown in Figure 4(e), miR-623 mimic transfection was observed to inhibit luciferase reporter activity in WT-transfected cells and not in mutated *TXNIP*-transfected cells. This indicated that *TXNIP* mRNA was directly targeted by miR-623. Subsequently, qRT-PCR and immunoblotting analysis revealed that miR-623 overexpression significantly downregulates mRNA and protein expression levels of TXNIP in NP cells (Figures 4(f) and 4(g)). To further confirm whether TXNIP expression is regulated by TBHP via miR-623, NP cells were transfected with miR-623 mimic and treated with TBHP. As shown in Figure 5(a), TBHP was found to induce TXNIP expression, while miR-623 overexpression was observed to alleviate this effect.

To assess the role of TXNIP and miR-623 in TBHP-induced apoptosis and inflammation, we cotransfected NP cells with miR-623 mimic and TXNIP. Immunoblotting analysis showed that miR-623 inhibits TBHP-induced expression of proapoptotic proteins such as BAX, BCL2, and cleaved caspase-3; inflammatory mediators including IL-1*β*, IL-6, and iNOS; and the majority of matrix degrading proteases such as MMP-13. In contrast, TXNIP was found to enhance the expression of these proapoptotic proteins, inflammatory mediators, and matrix-degrading proteases (Figures 5(b)–5(e)). These results suggested that miR-623 attenuates TBHP-induced apoptosis and inflammation by targeting *TXNIP* mRNA.

### 2.5. miR-623 Expression Is Regulated by HIF-1*α* under Oxidative Stress Conditions

**Figure 6:**
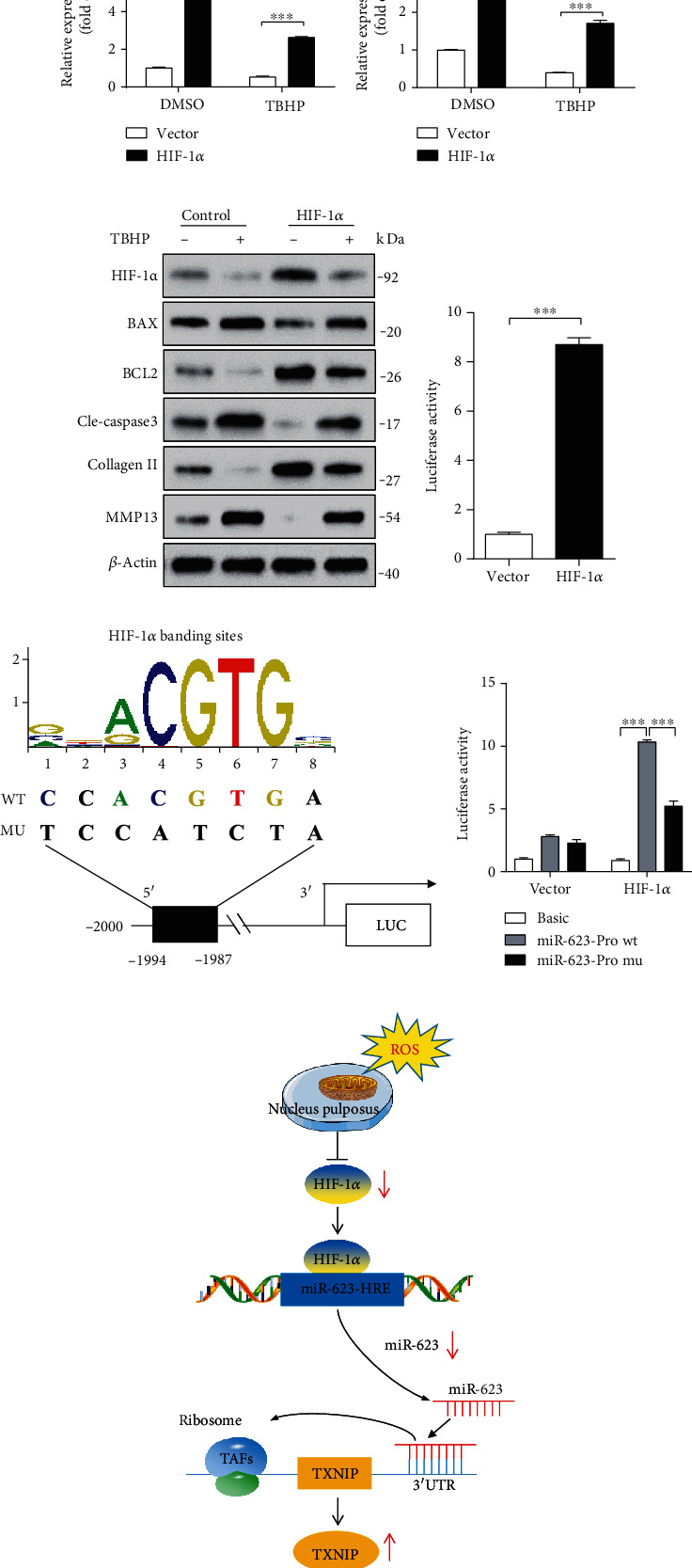
miR-623 expression is regulated by HIF-1*α* under oxidative stress. (a) Western blot analysis of HIF-1*α* expression in normal and degenerated NP tissues. (b, c) Quantification analysis of HIF-1*α* and miR-623 expression in NP cells induced by TBHP with or without HIF-1*α* overexpression. (d) Western blot analyses of HIF-1*α*, BAX, BCL2, cleaved caspase-3, collagen II, and MMP13 in NP cells induced by TBHP with or without HIF-1*α* overexpression. (e) miR-623 promoter activity induced by HIF-1*α* overexpression. (f) The schematic of the WT and mutant miR-623 promoter constructs. (g) miR-623 promoter activity induced by HIF-1*α* with or without putative HIF-1*α* binding site mutation on the miR-623 promoter. (h) The schematic graph reflects the HIF-1*α*-miR-623-TXNIP pathway in NP cell apoptosis and inflammatory responses induced by oxidative stress. Normally, NP is an avascular tissue under a hypoxic environment and expresses a high level of HIF-1*α*. Under oxidative stress conditions, HIF-1*α* expression is dampened and results in decreased nuclear translocation to bind to the hypoxia-reactive element on the miR-623 promoter, leading to decreased miR-623 transcription. The dampened miR-623 expression alleviates the inhibited effect miR-623 on TXNIP expression by targeting to its 3′-UTR, ultimately leading to increased apoptosis and inflammatory responses of NP cells and initiating the IVDD. ^∗∗∗^*P* < 0.001. *P* values were analyzed by two-tailed *t*-tests in (e) and two-way ANOVA in (b, g).

In IVDs, NP is an avascular tissue under the hypoxic environment. HIF is one of the vital factors that has been shown to directly mediate cellular responses to hypoxia [2]. In our previous study, we demonstrated the protective effect of HIF-1*α* against apoptosis in NP cells [2]. In the present study, results revealed that HIF-1*α* expression was significantly decreased in NP cells of IVDD tissue (Figure 6(a)). In addition, some studies have reported that oxidative stress inhibits HIF-1*α* in NP cells [14], and our present study further reveals that oxidative stress mediates oxidative stress-induced NP cell apoptosis and inflammatory responses via miR-623 (Figure 3). Therefore, we hypothesized that HIF-1*α* might be acting as an intermediator between oxidative stress and miR-623 expression in NP cells. To evaluate our hypothesis, we first detected miR-623 expression in oxidative stress-mediated NP cells with or without HIF-1*α* overexpression. Results showed that HIF-1*α* overexpression significantly restored miR-623 expression, which was downregulated by TBHP (Figures 6(b) and 6(c)). Consistent with miR-623 mimic-related results, HIF-1*α* overexpression was found to markedly restore the expression of proapoptotic proteins (BAX and cleaved caspase-3), antiapoptotic proteins (BCL2), and ECM metabolism-related proteins (MMP13 and collagen II), which were upregulated and downregulated, respectively, on TBHP treatment (Figure 6(d)).

HIF-1*α* is a transcriptional factor that usually binds to the promoter region of target genes to facilitate their expression [15]. Thus, we investigated whether HIF-1*α* mediates miR-623 expression by directly binding to its promoter region. A dual-luciferase reporter gene assay system was used to detect the promoter activity of miR-623 on HIF-1*α* overexpression. Results showed that consistent with the mRNA expression of HIF-1*α* and miR-623, the promoter activity of miR-623 was found to be significantly augmented on HIF-1*α* overexpression (Figure 6(e)). Further, we investigated the location of the hypoxia-reactive element (HRE) in the miR-623 promoter region. On analyzing the promoter sequence of human miR-623 using the JASPAR core database [16], we found one putative binding site for HIF-1*α* at −1,994/−1,987 bp (CCACGTGA) on its promoter region (Figure 6(f)). To further examine if the predicted binding site is an essential component for HIF-1*α*-regulated miR-623 expression, we mutated the putative binding sites to TCCATCTA (Figure 6(f)). NP cells were then transfected with either WT or mutated constructs, and luciferase activity was measured following HIF-1*α* overexpression. Results revealed that compared to WT controls, mutation in the binding site reduced the promoter activity of miR-623 induced by HIF-1*α* overexpression (Figure 6(g)). Overall, these results demonstrated that miR-623 expression was regulated by HIF-1*α* under oxidative stress conditions.

## 3. Discussion

Excessive apoptosis and inflammatory responses in NP cells induced by oxidative stress can trigger metabolic disorders of NP tissues, obliterate the normal structure and physiological functions of IVD, and eventually lead to IVDD [17, 18]. Although many miRNAs have been associated with IVDD, the specific miRNAs that can co-mediate apoptosis and the inflammatory response in oxidative stress-induced NP cells still need further identification. In this study, we found that miR-623 expression is downregulated in IVDD, which is regulated by HIF-1*α* under oxidative stress conditions. Furthermore, we demonstrated that miR-623 targets *TXNIP* mRNA and acts as an intermediator in apoptosis and inflammatory responses of oxidative stress-induced NP cells (Figure 6(h)). Thus, we proved miR-623 as an intermediator between oxidative stress and apoptosis and inflammatory responses of NP cells. Overall, on elucidating the expression and functional mechanisms of miR-623, our study suggests miR-623 as a therapeutic target for treating oxidative stress-induced IVDD.

IVDD has been reported to be typically instigated from the inner NP core, which has been shown to develop an altered cell phenotype with downregulated expression of proteoglycans and collagen II. Furthermore, studies have shown that such alterations can reduce pressure within the NP core and cause increased compressive stress towards the inner annulus, which has been demonstrated to trigger disc herniation [19]. Pathogenesis of IVDD has been shown to involve a complex signaling network and various effector molecules [20, 21]. Recent studies have reported that the onset and progression of IVDD is strictly associated with ROS and oxidative stress. Oxidative stress not only reinforces matrix degradation and inflammation but also promotes the decrease in the number of viable and functional cells in the IVD microenvironment. This has been observed to impair the mechanical function of IVDs and intensify the progression of IVDD [7]. Thus, evaluating oxidative stress-induced molecular mechanisms that mediate apoptosis and inflammatory responses in NP cells and finding an oxidative stress-targeted therapeutic strategy would provide a novel perspective in IVDD treatment. Our present study revealed that miR-623 expression was reduced in IVDD and regulated by HIF-1*α* under oxidative stress conditions. Furthermore, we showed that miR-623 acted as an intermediator of apoptosis and inflammatory responses in oxidative stress-induced NP cells by directly targeting 3′-UTR of *TXNIP* mRNA.

TXNIP is a type of thioredoxin-interacting protein from the *α*-suppressor protein family and is expressed in a variety of cells and tissues. Besides functioning as an initial tumor suppressor protein [22], TXNIP has also been shown as a key regulatory protein in oxidative stress-induced apoptosis and inflammatory responses [13]. Clusters of ROS have been demonstrated to promote nuclear translocation of TXNIP from cytoplasm and concurrently activate inflammation or apoptosis-related signaling molecules such as apoptosis signal-regulating kinase 1 (ASK1) and NACHT, LRR, and PYD domain-containing protein 3 (NLRP3) [23, 24]. Furthermore, upregulated expression of TXNIP has been shown to be accompanied by activation of inflammatory signals in IVDD [18]. In the present study, TXNIP expression was found to be upregulated in IVDD, which played a crucial role in apoptosis and inflammatory responses of oxidative stress-induced NP cells. Further, we found that miR-623 was a key regulator of TXNIP expression by directly targeting 3′-UTR of *TXNIP* mRNA.

In IVDs, NP is an avascular tissue under the hypoxic environment. As a cellular adaptation, alterations in the oxygen level have been reported to facilitate or inhibit HIF-1*α* activation. This mechanism has been shown to promote expression of diverse homeostasis regulatory genes that mediate cell survival and accommodation [25]. In our earlier study, we show in NP-specific HIF-1*α*-deficient mice that HIF-1*α* plays a vital role in the survival of NP cells and ECM homeostasis. However, mechanisms by which HIF-1*α* mediates cell survival are still not well elucidated [2]. In the present study, we found that HIF-1*α* expression was downregulated in NP cells under oxidative stress conditions. We also demonstrated that HIF-1*α* could mediate NP cell apoptosis induced by oxidative stress via miR-623. Moreover, we proved that HIF-1*α* facilitated miR-623 expression by directly binding to the promoter region of miR-623 at −1,994/−1,987 bp.

Although these results are promising, we would like to point out some potential limitations of this study. First, the majority of the experiments were performed *in vitro*, which might not essentially complement the *in vivo* mechanisms. Second, the human NP cell culture was not monitored under conditions of hypoxia, which is physiologically relevant and may affect cell growth. Third, in the spine, one vertebra could be regarded a single oncologic compartment, as cartilaginous endplate and cartilaginous annulus fibrosus served as strong barriers to spinal tumor spread [26, 27]. And, all lesions of the cases selected showed well-defined anatomic compartment and the tumors did not invade the intervertebral disc. Moreover, the NP tissues from spinal tumor patients with Pfirrmann grades I and II were used in the present study, which showed no obvious degenerative phenotypes. All of which indicated that these NP tissues could be regarded as the normal controls. However, it is still necessary to determine that miR-623 expression in NP tissues were not affected by the spinal tumor microenvironment, as it has been reported that miR-623 is a tumor suppressor and its expression may change in tumor patients [28–30]. Finally, although the essential role of the HIF-1*α*-miR-623-TXNIP pathway was observed in regulating apoptosis and inflammatory responses induced by oxidative stress in NP cells, it must be noted that the findings were collected from the experiment results within normal NP cells. Since there are degenerative cells in the degenerative disc, not normal cells, whether the responses of these two kinds of cells to the same stimulus are consistent still needs further investigation.

In conclusion, this study demonstrated that HIF-1*α*-induced miR-623 could regulate apoptosis and inflammation in TBHP-induced NP cells by targeting *TXNIP* mRNA. These findings improved our understandings of the mechanism involved in IVDD pathogenesis and might be a valuable resource to develop potentially effective therapeutic strategies against IVDD.

## 4. Materials and Methods

### 4.1. Bioinformatics Analysis

The miRNA expression profile datasets GSE63492 and GSE19943 were downloaded from the GEO database. The two-microarray expression data were compiled through Venn analysis, and the binding sites of HIF-1*α* were predicted via the JASPAR database (http://jaspar.binf.ku.dk/cgi-bin/jaspar_db.pl). The sequence of the miR-623 promoter was founded in the UCSC database (http://genome.ucsc.edu). Meanwhile, the gene expression profile dataset GSE34095 was downloaded from the GEO database. The predicted targets of miR-623 and the upregulated gene expression of GSE34095 were compiled. The mRNA targets of miR-623 were predicted using two programs: TargetScan (http://www.targetscan.org), miRTarBase (http://mirtarbase.mbc.nctu.edu.tw/php/download.php).

### 4.2. Ethics Statement

All procedures were approved by the Ethics Committee of Shanghai Changzheng Hospital, Second Military Medical University, China (no. 2017SL040), and were carried out only after written informed consent had been obtained from all study participants and from the parents of subjects younger than 18 years of age.

### 4.3. NP Tissue Collection

Before the operation, the patients were examined via MRI and IVD degeneration was evaluated according to the classification system described by Pfirrmann et al. [31]. The degenerative NP tissues were obtained from 11 patients undergoing intervertebral disc discectomy in Changzheng Hospital, with Pfirrmann grades III and V. And the control NP tissues were obtained from 11 patients with spinal tumors undergoing total spondylectomy and reconstruction, with Pfirrmann grades I and II. Characteristics of the patients are summarized in Tables 1 and 2.

### 4.4. NP Cell Culture

NP cells were isolated from NP tissues of spinal tumor patients, with Pfirrmann grade I. NPCs were cultured in DMEM/F12 (HyClone) with 10% fetal bovine serum. Second-passage cells were used in all experiments. NPCs were treated with 100 *μ*M TBHP (Sigma, MO, USA) for 12 h to induce oxidative stress.

### 4.5. RNA FISH

In situ hybridization was performed to detect the expression of miR-623 in NP tissues by using specific probes. Blue fluorescence indicated cell nucleus and, green fluorescence indicated miR-623.

### 4.6. Analysis of Cell Apoptosis

Cell apoptosis was detected by Annexin V-PI flow cytometry assay. NPs were transfected with miRNA mimics or mimic control followed by TBHP treatment as indicated. Annexin V FITC Apop Dtec Kit I (BD, 556547) was used for assessing cell apoptosis according to the manufacturer's instructions.

### 4.7. RNA Isolation and qRT-PCR Analysis

Total RNA was extracted from NP tissues using TRIzol (TaKaRa). (Invitrogen Corporation, 15,596–018) according to the manufacturer's instructions. The PrimeScript™ RT Reagent Kit (Perfect Real Time, TaKaRa, RR037A) and SYBR Premix EX TaqTM (TaKaRa, RR820A) were used to detect and quantify miR-623, and RNU6/U6 was used as an internal control. For mRNA studies, The PrimeScript™ RT Master Mix (Perfect Real Time, TaKaRa, RR036A) was used to reverse transcript and RT-PCR was performed to detect HIF-1*α* and TXNIP levels by using SYBR Premix EX Taq TM (TaKaRa, RR820A). GAPDH (glyceraldehyde-3-phosphate dehydrogenase) served as the reference gene. Relative expression was calculated using the comparative threshold cycle (Ct) method. Experiments were carried out in triplicate. A complete list of primers used was shown in Table 3.

### 4.8. Western Blotting

Protein concentration was determined by a BCA kit (KeyGen, KGP902). Protein extracts were then separated by electrophoresis in 10–12% polyacrylamide gels and were transferred to poly-vinylidene difluoride membranes (Millipore Sigma, IPVH00010). After blocking in 5% skim milk (Biofroxx, 1172GR500), the membranes were incubated with the indicated primary antibodies (Bax: #2772, CST; Bcl2: #4223, CST; cle-caspase3: #9664, CST; TXNIP: #14715, CST; iNOS: #13120, CST; HIF-1*α*: #36169, CST; IL-1*β*: #12703, CST; Collagen II: ab34712, Abcam; MMP13: ab219620, Abcam; *β*-action: #4970, CST; and cleaved caspase-1: #4199, CST) at 4°C overnight, following by the corresponding horseradish peroxidase-conjugated secondary antibodies (anti-mouse IgG, HRP-linked antibody: #7076, CST; anti-rabbit IgG, HRP-linked antibody: #7074, CST). Signals were detected using chemiluminescent ECL reagent (Cytiva, RPN2235). Both the primary antibody and the second antibody were diluted in 1 : 1000.

### 4.9. Detection of the Mitochondrial Membrane Potential

The mitochondrial membrane potential was detected by a JC-1 kit (C2006; Beyotime, China) according to the manufacturer's instructions. Briefly, the NP cells were collected and resuspended in 1 mL of JC-1 staining buffer and then incubated in the dark at 37°C for 20 min, centrifuged for the collection of cell precipitation, washed with JC-1 staining buffer (1×) twice, 500 *μ*l JC-1 staining buffer (1×), and detected by flow cytometry.

### 4.10. ELISA

The supernatants of cell culture were collected for cytokine evaluation. Cytokine production was measured by human IL-1*β* and IL-6 Quantikine ELISA Kit (R&D Systems, DLB50 and D6050) according to the manufacturer's instructions.

### 4.11. Lipid ROS Assay

BODIPY-C11 dye (Thermo, D3861) was used to detect the lipid ROS level. Briefly, add BODIPY-C11 to the culture medium for the 5 *μ*M final concentration of BODIPY-C11. The culture was returned to the cell culture incubator for 20 min. Cells were collected in 1.5 mL tubes and washed twice with PBS. The amount of ROS within cells was determined by flow cytometry using the Beckman CyAn AOP.

### 4.12. Luciferase Reporter Assay

The promoter miR-623 luciferase reporter constructs containing the wild-type binding sites of HIF-1*α* were amplified using the PCR method. The PCR products were cloned into the pGL3-report luciferase vector, immediately upstream of the luciferase gene. All the constructs containing promoter inserts were sequenced and verified.

The 3′-UTR-TXNIP luciferase reporter constructs containing the wild-type and mutant binding sites of miR-623 were amplified using the PCR method. The PCR products were cloned into the pMiR-report luciferase vector (Ambion, AM5795), immediately downstream of the luciferase gene. All the constructs containing 3′-UTR inserts were sequenced and verified.

Cells were cotransfected with reporter constructs, and the miRNA mimic and the *β*-gal plasmid were harvested 48 h after the transfection and lysed with reporter lysis buffer (Promega, E397A). The luciferase activities in the cellular extracts were determined using the dual-luciferase reporter assay system (Promega, E1910) according to the manufacturer's instructions. Data were represented as the fold induction after normalizing the luciferase activity of the tested sample to that of the corresponding control sample.

### 4.13. Statistical Analysis

All data representative of three independent experiments are present as mean ± SEM. We used two-tailed *t*-tests to determine significances between two groups. We did analyses of multiple groups by one- or two-way ANOVA with Bonferroni post-test of GraphPad prism version 5. For all statistical tests, we considered a *P* value < 0.05 to be statistically significant.

## Figures and Tables

**Table 1:** Characteristics of the patients with disc degeneration.

Patient no.	Sex	Age	Diagnosis	Sample level	Disc of Pfirrmann grade
1	Female	17	Intervertebral disc herniation	L4-L5	V
2	Male	26	Intervertebral disc herniation	L4-L5	V
3	Female	65	Intervertebral disc herniation	L4-L5	III
4	Male	56	Intervertebral disc herniation	L4-L5	IV
5	Male	21	Intervertebral disc herniation	L5-S1	V
6	Female	32	Intervertebral disc herniation	L4-L5	IV
7	Female	64	Intervertebral disc herniation	L4-L5	IV
8	Female	62	Intervertebral disc herniation	L3-L4	IV
9	Male	74	Intervertebral disc herniation	L4-L5	V
10	Male	26	Intervertebral disc herniation	L3-L4	IV
11	Female	56	Intervertebral disc herniation	L4-L5	V

**Table 2:** Characteristics of the patients with spinal tumor.

Patient no.	Sex	Age	Diagnosis	Sample level	Disc of Pfirrmann grade
1	Female	34	L4 giant cell tumor	L4-5	I
2	Male	23	L3 osteosarcoma	L3-4	II
3	Male	25	C6 giant cell tumor	C6-7	I
4	Male	32	L1 chondrosarcoma	L1-2	I
5	Female	56	T12 Ewing's sarcoma	T12-L1	I
6	Female	45	L1 metastatic breast cancer	L1-2	I
7	Female	25	T11 metastatic renal cancer	T11-12	I
8	Male	43	T10 giant cell tumor	T10-11	II
9	Male	25	L4 metastatic lung cancer	L3-4	I
10	Female	36	T8 solitary plasmacytoma	T7-8	I
11	Male	43	C5 osteoblastoma	C4-5	I

**Table 3:** Primer sequences for real-time PCR.

Gene	Gene ID		Primer sequence(5′-3′)
GAPDH	2597	Forward	TCCACTGGCGTCTTCACC
		Reverse	GGCAGAGATGATGACCCTTTT
IL-6	3569	Forward	ACTCACCTCTTCAGAACGAATTG
		Reverse	CCATCTTTGGAAGGTTCAGGTTG
TXNIP	10628	Forward	TGTGTGAAGTTACTCGTGTCAAA
		Reverse	GCAGGTACTCCGAAGTCTGT
IL-1*β*	3553	Forward	CGAATCTCCGACCACCACTAC
		Reverse	TCCATGGCCACAACAACTG
HIF-1*α*	3091	Forward	CCACTGCCACCACTGATGAA
		Reverse	GTGAGGCTGTCCGACTTTGA
RNU6	26827	Forward	CTCGCTTCGGCAGCACA
		Reverse	AACGCTTCACGAATTTGCGT
miR-623	693208	Forward	ATCCCTTGCAGGGGCTGTTGGGT
		Reverse	AACGCTTCACGAATTTGCGT

## Data Availability

The data that support the findings of this study are available from the corresponding author upon reasonable request.
